# Risk factors for intraocular pressure elevation in eyes with intraocular lens subluxation or dislocation

**DOI:** 10.1007/s00417-025-06774-0

**Published:** 2025-02-15

**Authors:** Kentaro Iwasaki, Shogo Arimura, Marie Suzuki, Yoshihiro Takamura, Masaru Inatani

**Affiliations:** Department of Ophthalmology, Faculty of Medical Sciences, University of Fukui, 23-3 shimoaizuki, Matsuoka, Eiheiji, Yoshida, Fukui, 910-1193 Japan

**Keywords:** Risk factor, IOP elevation, IOL displacement, IOL subluxation, IOL dislocation, Exfoliation, Glaucoma

## Abstract

**Purpose:**

To investigate the risk factors for intraocular pressure (IOP) elevation in eyes with intraocular lens (IOL) subluxation or dislocation.

**Methods:**

We retrospectively examined the eyes with IOL displacement (either IOL subluxation or IOL dislocation) who underwent IOL refixation combined with vitrectomy between September 1, 2012, and May 31, 2024, at Fukui University Hospital. Patients were divided into two groups: those with IOL subluxation and those with IOL dislocation. Additionally, subgroups were created for eyes without glaucoma and those without both glaucoma and exfoliation syndrome. IOL subluxation was defined as the movement of the IOL–capsular bag complex in the posterior chamber, while IOL dislocation was defined as a fall of the IOL–capsular bag complex into the vitreous space. Risk factors for preoperative IOP elevation in eyes with IOL displacement were identified using multivariate analysis with a multiple linear regression model.

**Results:**

This study included 155 eyes with IOL displacement (IOL subluxation; 73 eyes and IOL dislocation; 82 eyes). Multivariate analyses revealed that IOL subluxation and the number of glaucoma medications were significantly associated with a higher preoperative IOP (*P* < 0.01). The preoperative IOP and the number of glaucoma medications were significantly higher in the IOL subluxation group than in the IOL dislocation group (22.7 ± 9.0 vs. 15.5 ± 3.9 mmHg, *P* < 0.01, and 1.2 ± 1.7 vs. 0.2 ± 0.8, *P* < 0.01, respectively). The number of patients with exfoliation syndrome and glaucoma was significantly higher in the IOL subluxation group than in the IOL dislocation group (49% vs. 10%, *P* < 0.01, and 25% vs. 7%, *P* < 0.01, respectively). In the subgroup analysis in eyes without glaucoma (131 eyes), IOL subluxation and the number of glaucoma medications were significantly associated with a higher preoperative IOP (*P* < 0.01). In this subgroup, the preoperative IOP and the number of glaucoma medications were significantly higher in the IOL subluxation group than in the IOL dislocation group (20.6 ± 8.5 vs. 15.5 ± 3.9 mmHg, *P* < 0.01, and 0.4 ± 1.0 vs. 0.04 ± 0.3, *P* < 0.01, respectively), and the incidence of exfoliation syndrome was significantly higher in the IOL subluxation group than in the IOL dislocation group (40% vs. 8%, *P* < 0.01). In another subgroup analysis in eyes without glaucoma and exfoliation syndrome (103 eyes), IOL subluxation and the number of glaucoma medications were significantly associated with a higher preoperative IOP (*P* < 0.01). In this subgroup, the preoperative IOP and the number of glaucoma medications were significantly higher in the IOL subluxation group than in the IOL dislocation group (19.3 ± 7.3 vs. 15.7 ± 4.0 mmHg, *P* = 0.049, and 0.3 ± 0.9 vs. 0.0 ± 0.0, *P* < 0.01, respectively).

**Conclusions:**

IOL subluxation, but not dislocation, is a risk factor for elevated IOP in eyes with IOL displacement.

## Abstract (highlights)

**Key messages:**

*****What is known***:**

- Intraocular pressure (IOP) elevation occurs and requires treatment in eyes with intraocular lens (IOL) displacement, however, the risk factors for IOP elevation in eyes with IOL displacement remain unclear.

*****What is new***:**

- IOL subluxation, but not dislocation, is a risk factor for elevated IOP in eyes with IOL displacement.

## Introduction

After cataract surgery, a progressive loosening of zonules can occur over time [1, 2]. In particular, eyes with exfoliation syndrome are more prone to develop weak zonules [1, 3, 4]. Exfoliation syndrome is a well-known risk factor for glaucoma and subluxated intraocular lens (IOL) [1–3, 5–7]. In many previous studies, IOL refixation surgery combined with different glaucoma surgeries was performed to control elevated intraocular pressure (IOP) in cases of glaucoma eyes with IOL displacement (IOL subluxation or dislocation), including exfoliation syndrome [8–14]. Those studies demonstrated that IOL refixation combined with glaucoma surgery was effective for IOP control in patients with IOP elevation due to IOL displacement. Some studies have suggested that correction of IOL displacement alone decreases IOP in eyes with IOP elevation due to IOL displacement [15–18]. Thus, it is well known that IOP elevation occurs and requires treatment in eyes with IOL displacement.

Several hypotheses have been proposed to explain IOP elevation in eyes with IOL displacement. Changes in the anatomy of the anterior segment and prolapse of the anterior vitreous may affect aqueous flow, which subsequently increases IOP [17]. Uveitis-glaucoma-hyphema (UGH) syndrome has also been considered as a possible mechanism [8]. This syndrome can be related to a mobile IOL capsular bag complex, resulting in anterior axial movement and intermittent uveal chafing by the IOL edge within the capsular bag [19, 20]. Pigment overload or inflammation may cause reduction of the aqueous outflow facility with elevated IOP. However, there are cases where IOP increases and cases in which it does not occur in eyes with IOL displacement. The risk factors for IOP elevation in eyes with IOL displacement remain unclear. Therefore, this study aims to evaluate these risk factors.

## Methods

### Patient selection

This is a retrospective study in which we included the patients operated between September 1, 2012, and May 31, 2024, at the Fukui University Hospital. The included patients had IOL displacement (IOL subluxation or dislocation) and had previously undergone IOL refixation combined with vitrectomy. IOL subluxation was defined as the movement of the IOL–capsular bag complex in the posterior chamber, while IOL dislocation was defined as a fall of the IOL–capsular bag complex into the vitreous space. The IOL refixation was performed using the flanged fixation [21], T-fixation [22], or sulcus fixation [23] technique. We excluded patients aged < 20 years and those who had undergone previous IOL refixation surgery. We evaluated the eyes that were treated first in cases in which both eyes of the same patient fulfilled the study criteria. Patients were divided into two groups: those with IOL subluxation and those with IOL dislocation. Additionally, subgroups were created for eyes without glaucoma and those without both glaucoma and exfoliation syndrome. Glaucoma refers to an eye that was originally treated for glaucoma and had glaucomatous optic neuropathy based on funduscopic observation preoperatively. Risk factors for preoperative IOP elevation in eyes with IOL displacement were identified using multivariate analysis with a multiple linear regression model.

### Outcome measures and data collection

We collected data on age, sex, IOL subluxation or dislocation, type of glaucoma, previous intraocular surgery, preoperative IOP, and the number of glaucoma medications. Preoperative data were obtained from the patient’s medical records. Risk factors for IOP elevation in eyes with IOL subluxation or dislocation were the main outcome measures.

#### Statistical analysis

The chi-square test, Fisher’s exact test, and Mann–Whitney *U* nonparametric test were used to perform univariate comparisons between the groups. Stepwise multiple linear regression analysis was performed to determine variables associated with preoperative IOP. A *P*-value < 0.05 was considered statistically significant. The SPSS software (version 24.0; IBM Institute, Inc. Chicago, IL, USA) was used for statistical analysis.

## Results

### Patient characteristics

This study included 155 eyes from 155 patients with IOL displacement who underwent IOL refixation combined with vitrectomy. Table 1 summarizes the patients’ preoperative characteristics. Among the 155 eyes, 24 (15%) were originally treated for glaucoma. Eight eyes (5%) had previously undergone glaucoma surgery, including ab interno trabeculotomy using Kahook dual blade, trabeculectomy or Ahmed glaucoma valve. The thirty three eyes (21%) had previously undergone vitrectomy for retinal detachment, macular hole, epiretinal membrane or vitreous hemorrhage.

The patients were divided into two groups: those with IOL subluxation (*n* = 73) and those with IOL dislocation (*n* = 82). Table 1 also shows a comparison between the two groups. Preoperative IOP was significantly higher in the IOL subluxation group (22.7 ± 9.0) than in the IOL dislocation group (15.5 ± 3.9) (*P* < 0.01). The number of patients with preoperative IOP > 21 mmHg was significantly higher in the IOL subluxation group (48%) than in the IOL dislocation group (2%) (*P* < 0.01). The incidence of exfoliation syndrome was significantly higher in the IOL subluxation group (49%) than in the IOL dislocation group (10%) (*P* < 0.01). The number of patients with glaucoma was significantly higher in the IOL subluxation group (25%) than in the IOL dislocation group (7%) (*P* < 0.01). Additionally, the number of glaucoma medications was significantly higher in the IOL subluxation group (1.2 ± 1.7) than in the IOL dislocation group (0.2 ± 0.8) (*P* < 0.01). No other significant differences were observed between the two groups.

**Table 1:** Patient characteristics

	Total (*n* = 155)	IOL subluxation (*n* = 73)	IOL dislocation (*n* = 82)	*P*-value
Age, years	72.4 ± 14.5	73.9 ± 14.5	71.1 ± 14.4	0.16
Sex, *n* (%)				0.33
Male	92 (59)	40 (55)	52 (63)	
Female	63 (41)	33 (45)	30 (37)	
Preoperative IOP, mmHg	18.9 ± 7.7	22.7 ± 9.0	15.5 ± 3.9	< 0.01
Preoperative IOP > 21 mmHg, *n* (%)	37 (24)	35 (48)	2 (2)	< 0.01
Exfoliation syndrome, *n* (%)	44 (28)	36 (49)	8 (10)	< 0.01
Glaucoma, *n* (%)	24 (15)	18 (25)	6 (7)	< 0.01
Type of glaucoma, *n* (%)				0.08
Exfoliation glaucoma	16 (67)	14 (77)	2 (33)	
Primary open-angle glaucoma	5 (21)	2 (11)	3 (50)	
Primary angle closure glaucoma	1 (4)	1 (6)	0 (0)	
Neovascular glaucoma	1 (4)	0 (0)	1 (17)	
Uveitis glaucoma	1 (4)	1 (6)	0 (0)	
Glaucoma medications, *n*	0.6 ± 1.4	1.2 ± 1.7	0.2 ± 0.8	< 0.01
Previous glaucoma surgery, *n* (%)	8 (5)	3 (4)	5 (6)	0.72
Previous vitrectomy, *n* (%)	33 (21)	12 (16)	21 (26)	0.18

Data shown as mean ± standard deviation

*IOL* intraocular lens, *IOP* intraocular pressure

*P* values were calculated comparing between IOL subluxation and IOL dislocation

### Outcome measure

Age, IOL subluxation or dislocation, glaucoma status, exfoliation syndrome status, the number of glaucoma medications, previous glaucoma surgery, and previous vitrectomy were evaluated as possible determinants of preoperative IOP. Multivariate analyses using stepwise multiple linear regression models in all eyes demonstrated that IOL subluxation and the number of preoperative glaucoma medications were significantly associated with higher preoperative IOP (Table 2). Among the two factors, the number of glaucoma medications had the highest standardized coefficients (Beta = 0.39). No other statistically significant determinants were identified.

**Table 2:** Multivariate analysis for determining variables associated with the higher preoperative IOP using a multiple regression model in all eyes (*n* = 155)

Variable	Unstandardized coefficients B	Standardized coefficients Beta	T	*P*-value
(Constant)	15.14		21.88	< 0.01
The number of glaucoma medications	2.14	0.39	5.57	< 0.01
IOL subluxation / IOL dislocation	5.01	0.33	4.66	< 0.01

*IOL* intraocular lens

The incidence of exfoliation syndrome and the number of patients with glaucoma were significantly higher in the IOL subluxation group than in the IOL dislocation group. The glaucoma and exfoliation syndrome could be contributing to the higher preoperative IOP. Therefore, subgroup analyses were performed in the glaucoma exclusion group (*n* = 131) and the glaucoma and exfoliation syndrome exclusion group (*n* = 103). Multivariate analyses were performed using stepwise multiple linear regression models in eyes without glaucoma (*n* = 131) (Table 3). Age, IOL subluxation or dislocation, exfoliation syndrome status, the number of glaucoma medications, and previous vitrectomy were evaluated as possible determinants of preoperative IOP. In this subgroup, IOL subluxation and the number of preoperative glaucoma medications were significantly associated with a higher preoperative IOP. Among the two factors, IOL subluxation had the highest standardized coefficients (Beta = 0.31). No other statistically significant determinants were identified. Table 4 shows a comparison between the IOL subluxation (*n* = 55) and IOL dislocation (*n* = 76) groups in the eyes without glaucoma. Preoperative IOP was significantly higher in the IOL subluxation group (20.6 ± 8.5) than in the IOL dislocation group (15.5 ± 3.9) (*P* < 0.01). The number of patients with preoperative IOP > 21 mmHg was significantly higher in the IOL subluxation group (35%) than in the IOL dislocation group (3%) (*P* < 0.01). The incidence of exfoliation syndrome was significantly higher in the IOL subluxation group (40%) than in the IOL dislocation group (8%) (*P* < 0.01). Additionally, the number of glaucoma medications was significantly higher in the IOL subluxation group (0.4 ± 1.0) than in the IOL dislocation group (0.04 ± 0.3) (*P* < 0.01). No other statistically significant differences were observed between the groups.

**Table 3:** Multivariate analysis for determining variables associated with a high preoperative IOP using a multiple regression model in eyes without glaucoma (*n* = 131)

Variable	Unstandardized coefficients B	Standardized coefficients Beta	T	*P*-value
(Constant)	15.45		21.95	< 0.01
IOL subluxation / IOL dislocation	4.23	0.31	3.79	< 0.01
The number of glaucoma medications	2.16	0.22	2.69	< 0.01

*IOL* intraocular lens

**Table 4:** Comparison of patient characteristics in eyes with IOL subluxation and dislocation but without glaucoma

	IOL subluxation (*n* = 55)	IOL dislocation (*n* = 76)	*P*-value
Age, years	75.1 ± 15.1	71.3 ± 14.2	0.92
Sex, n (%)			0.47
Male	31 (56)	48 (63)	
Female	24 (44)	28 (37)	
Preoperative IOP, mmHg	20.6 ± 8.5	15.5 ± 3.9	< 0.01
Preoperative IOP > 21 mmHg, *n* (%)	19 (35)	2 (3)	< 0.01
Exfoliation syndrome, *n* (%)	22 (40)	6 (8)	< 0.01
Glaucoma medications, *n*	0.4 ± 1.0	0.04 ± 0.3	< 0.01
Previous vitrectomy, *n* (%)	12 (22)	19 (25)	0.84

Data shown in mean ± standard deviation

*IOL* intraocular lens, *IOP* intraocular pressure

Multivariate analyses were performed using stepwise multiple linear regression models in the eyes without glaucoma and exfoliation syndrome (*n* = 103) (Table 5). Age, IOL subluxation or dislocation, the number of glaucoma medications, and previous vitrectomy were evaluated as possible determinants of preoperative IOP. In this subgroup, IOL subluxation and the number of preoperative glaucoma medications were significantly associated with a higher preoperative IOP. Among the two factors, the number of glaucoma medications had the highest standardized coefficients (Beta = 0.25). No other statistically significant determinants were identified. Table 6 shows a comparison between the IOL subluxation (*n* = 33) and IOL dislocation (*n* = 70) groups in eyes without glaucoma and exfoliation syndrome. Age was significantly lower in the IOL subluxation group (63.2 ± 13.8) than in the IOL dislocation group (71.4 ± 14.1) (*P* < 0.01). Preoperative IOP was significantly higher in the IOL subluxation group (19.3 ± 7.3) than in the IOL dislocation group (15.7 ± 4.0) (*P* = 0.049). The number of patients with preoperative IOP > 21 mmHg was significantly higher in the IOL subluxation group (27%) than in the IOL dislocation group (3%) (*P* < 0.01). Additionally, the number of glaucoma medications was significantly higher in the IOL subluxation group (0.3 ± 0.9) than in the IOL dislocation group (0.0 ± 0.0) (*P* < 0.01). No other statistically significant differences were observed between the groups.

**Table 5:** Multivariate analysis for determining variables associated with a high preoperative IOP using a multiple regression model in eyes without glaucoma and exfoliation syndrome (*n* = 103)

Variable	Unstandardized coefficients B	Standardized coefficients Beta	T	*P*-value
(Constant)	15.73		25.73	< 0.01
The number of glaucoma medications	2.69	0.25	2.54	0.01
IOL subluxation / IOL dislocation	2.68	0.23	2.36	0.02

IOL, intraocular lens

**Table 6:** Comparison of patient characteristics in eyes with IOL subluxation and dislocation but without glaucoma and exfoliation syndrome

	IOL subluxation (*n* = 33)	IOL dislocation (*n* = 70)	*P*-value
Age, years	63.2 ± 13.8	71.4 ± 14.1	< 0.01
Sex, *n* (%)			0.50
Male	24 (73)	45 (64)	
Female	9 (27)	25 (36)	
Preoperative IOP, mmHg	19.3 ± 7.3	15.7 ± 4.0	0.049
Preoperative IOP > 21 mmHg, *n* (%)	9 (27)	2 (3)	< 0.01
Glaucoma medications, *n*	0.3 ± 0.9	0.0 ± 0.0	< 0.01
Previous vitrectomy, *n* (%)	10 (30)	18 (26)	0.64

Data shown in mean ± standard deviation

*IOL* intraocular lens, *IOP* intraocular pressure

## Discussion

Previous studies have shown that IOL re-fixation combined with glaucoma surgery [8–14] or correction of IOL displacement alone [15–18] is effective for IOP control in patients with IOP elevation due to IOL displacement. Therefore, it is well known that eyes with IOL displacement will experience IOP elevation and require some treatment [8–18]. However, in eyes with IOL displacement, there are cases in which the IOP increases and cases in which it does not increase. This study is unique in examining 155 eyes with IOL displacement and evaluating the risk factors for IOP elevation.

In some previous reports, the terms IOL dislocation and IOL subluxation have been used separately [8.9, 15–18], while in other reports the term dislocation has been used inclusive of subluxation [10–14]. We believe that the intraocular pathological conditions change between IOL dislocation and IOL subluxation. Therefore, we distinguished between IOL subluxation and IOL dislocation in this study. We used the term IOL displacement to include IOL dislocation and IOL subluxation.

Our findings showed that IOL subluxation was associated with higher preoperative IOP in both total and subgroup cases. Previous studies have suggested an association between late in-the-bag IOL displacement and increased IOP [15–18]. Those studies proposed various hypotheses regarding IOP elevation in eyes with IOL displacement. First, changes in the anatomy of the anterior segment and prolapse of the anterior vitreous may affect aqueous flow, which subsequently increases IOP [17]. However, if this hypothesis was correct, IOP elevation would occur in eyes with IOL dislocation, which would be inconsistent with our results. Second, UGH syndrome has also been considered as a potential mechanism [8]. This may be related to a mobile IOL capsular complex, causing anterior axial translation and intermittent uveal abrasions by the IOL edge within the capsule [19, 20]. As in uveitis glaucoma, pigmentary glaucoma, or exfoliation glaucoma, pigment overload, or inflammation in the anterior chamber may cause a reduction in the aqueous outflow facility with elevated IOP. When IOL dislocation occurs, pigment dispersion and inflammation in the anterior chamber are thought to decrease as compared to when IOL subluxation occurs. Therefore, UGH syndrome appears to occur in eyes with IOL subluxation but not in eyes with IOL dislocation. However, we could not evaluate the diagnosis of UGH syndrome or the level of preoperative inflammation, which are limitations of our study. Our study also showed that the number of glaucoma medications was associated with higher preoperative IOP in both total and subgroup cases. The use of glaucoma medications might have indicated medically uncontrollable higher preoperative IOP or higher number of patients with glaucoma. Indeed, preoperative IOP, the number of patients with glaucoma, and the number of glaucoma medications were significantly higher in the IOL subluxation group than in the IOL dislocation group in total cases (Table 1). Furthermore, in the subgroup analyses about the glaucoma exclusion group (Table 4) and the glaucoma and exfoliation syndrome exclusion group (Table 5), preoperative IOP and the number of glaucoma medications were significantly higher in the IOL subluxation group than in the IOL dislocation group. Therefore, these results suggest that glaucoma medications and preoperative elevated IOP are associated in the IOL subluxation group.

In eyes with IOL displacement IOP could increase because of exfoliative material, especially in eyes with exfoliation syndrome [1, 3, 4, 24–27]. However, exfoliation syndrome was not identified as a risk factor for a higher preoperative IOP in this study (Tables 2 and 3). The incidence of exfoliation syndrome was significantly higher in the IOL subluxation group than in the IOL dislocation group (Tables 1 and 4), so we performed a subgroup analysis to exclude the overlapping effects between exfoliation syndrome and IOL subluxation on preoperative IOP. Even in cases without either exfoliation syndrome or glaucoma, exfoliation syndrome was not identified as a risk factor for a higher preoperative IOP (Table 5), and the preoperative IOP was significantly higher in the IOL subluxation group than in the IOL dislocation group (Table 6). This result supports the idea that IOL subluxation is more associated with high preoperative IOP than with exfoliation syndrome.

This study had certain limitations owing to its retrospective nature. First, there may have been a selection bias in patient selection. In eyes with IOL subluxation, IOL refixation surgery is primarily selected for those with elevated IOP, and those without elevated IOP may be placed under observation. Therefore, it is possible that the IOL subluxation group included preferentially eyes with elevated IOP. Second, we could not collect clinical data regarding inflammation in the anterior chamber and the condition of the subluxated IOL. Inflammation in the anterior chambers may have affected IOP elevation because of increased resistance to aqueous outflow [28]. Measurement of flare values using a flare cell meter or the measurement of inflammatory cytokines in the aqueous humor may have solved this problem. Evaluation of the condition of the subluxated IOL using anterior segment optical coherence tomography or ultrasound biomicroscopy may have revealed the cause of IOP elevation (e.g., UGH syndrome). Additional prospective studies, including all eyes with non-operated IOL displacement, are needed to address these limitations.

In conclusion, IOL subluxation is a risk factor for elevated IOP in eyes with IOL displacement. IOP elevation was unlikely to occur in eyes with IOL dislocation. Therefore, we must be more careful about IOP elevation in eyes with IOL subluxation.

## Data Availability

The datasets generated during and/or analyzed during the current study are available from the corresponding author on reasonable request.
